# Auditory Multi-Stability: Idiosyncratic Perceptual Switching Patterns, Executive Functions and Personality Traits

**DOI:** 10.1371/journal.pone.0154810

**Published:** 2016-05-02

**Authors:** Dávid Farkas, Susan L. Denham, Alexandra Bendixen, Dénes Tóth, Hirohito M. Kondo, István Winkler

**Affiliations:** 1 Institute of Cognitive Neuroscience and Psychology, Research Centre for Natural Sciences, Hungarian Academy of Sciences, Budapest, Hungary; 2 Department of Cognitive Science, Faculty of Natural Sciences, Budapest University of Technology and Economics, Budapest, Hungary; 3 Cognition Institute and School of Psychology, University of Plymouth, Plymouth, United Kingdom; 4 School of Natural Sciences, Chemnitz University of Technology, Chemnitz, Germany; 5 Human Information Science Laboratory, NTT Communication Science Laboratories, NTT Corporation, Atsugi, Japan; University of Salamanca- Institute for Neuroscience of Castille and Leon and Medical School, SPAIN

## Abstract

Multi-stability refers to the phenomenon of perception stochastically switching between possible interpretations of an unchanging stimulus. Despite considerable variability, individuals show stable idiosyncratic patterns of switching between alternative perceptions in the auditory streaming paradigm. We explored correlates of the individual switching patterns with executive functions, personality traits, and creativity. The main dimensions on which individual switching patterns differed from each other were identified using multidimensional scaling. Individuals with high scores on the dimension explaining the largest portion of the inter-individual variance switched more often between the alternative perceptions than those with low scores. They also perceived the most unusual interpretation more often, and experienced all perceptual alternatives with a shorter delay from stimulus onset. The ego-resiliency personality trait, which reflects a tendency for adaptive flexibility and experience seeking, was significantly positively related to this dimension. Taking these results together we suggest that this dimension may reflect the individual’s tendency for exploring the auditory environment. Executive functions were significantly related to some of the variables describing global properties of the switching patterns, such as the average number of switches. Thus individual patterns of perceptual switching in the auditory streaming paradigm are related to some personality traits and executive functions.

## Introduction

The phenomenon of perception stochastically switching back and forth between possible interpretations of an unchanging stimulus is termed multi-stable perception (often referred as bi-stability, for a review, see [1]). For example, in the duck-rabbit illusion [2], a perceiver can see either a duck or a rabbit, and his/her perception can change over time between the two alternatives. Aafjes, Hueting, and Visser [3] were the first to show that individuals differ in how often they switch between the alternative percepts. Extending this observation, Denham et al. [4] found idiosyncratic switching patterns in a multi-stable auditory stimulus paradigm (auditory streaming [5]). These authors also found that listeners retained their idiosyncratic switching pattern even with over a year between successive tests. These observations suggest that the perception of ambiguous sound sequences can reveal individual differences in information processing. The current study explores whether the idiosyncratic patterns that characterize perception correlate with other more widely recognized differences in executive functions, personality traits, and creativity.

Individual differences in switching rate have been found in a number of visual multi-stable perceptual paradigms, such as binocular rivalry [3,6,7], structure-from-motion illusion [8], visual plaids, and reversible figures [9]. In the auditory modality, individual switching rate differences have been demonstrated for verbal transformations [9,10] and auditory streaming [9–11]. Some of the individual variability in switching rates have been linked to genetic markers [7], differences in brain activations [10,11], and inter-hemispheric connectivity [8].

**Fig 1:**
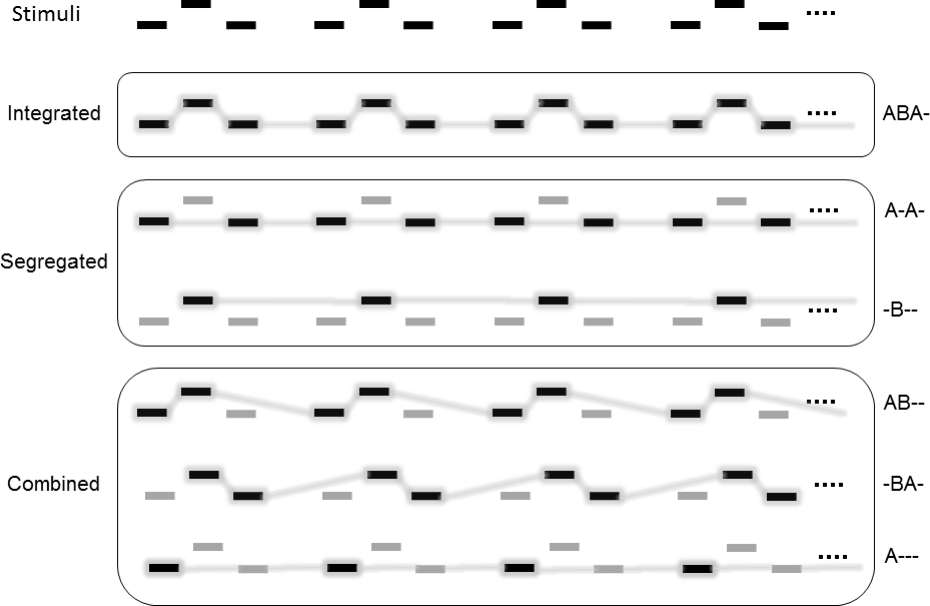
Schematic depiction of the auditory streaming paradigm (top panel) and its possible perceptual interpretations grouped into 3 categories (the 3 lower panels). Rectangles depict the “A” and “B” sounds with the feature difference between them indicated by displacement in the vertical direction. Time flows along the horizontal direction. Sounds perceived as part of the same stream are connected by lines in the lower panels. Darker notes with grey background indicate the stream in the foreground (also described with symbols to the right of each of the lower panels).

The auditory streaming paradigm [5] (Fig 1, top panel) has been widely used to study how the human auditory system separates concurrently active sound sources (termed auditory scene analysis by [12]). This stimulus consists of sequences of sounds of the form ABA-ABA-…, where “A” and “B” denote two different sounds and “-”stands for a silent interval with the same duration as A and B. Listeners mostly perceive this stimulus as either a single coherent sequence (or “stream”) consisting of the repeating ABA pattern with a characteristic galloping rhythm (termed the “integrated percept”; Fig 1, second panel) or as two separate isochronous streams in parallel, one consisting of the A, the other of the B sounds alone (termed the “segregated percept”; Fig 1, third panel). However, when given the option, from time to time, listeners also report other repeating patterns (together these are interchangeably termed the “both percept” or “combined percept” [4,13], we use the latter term throughout this manuscript; Fig 1, bottom panel). Perception of the “ABA-”sound sequence is influenced by the similarity of the A and B sounds (e.g. differences in pitch, location, timbre, etc.) and the presentation rate (for a review see [14]). Given sufficient time, perception has been shown to inevitably switch back and forth between the alternative percepts [15–22] even for stimulus parameter combinations that have been assumed to strongly promote one of the perceptual alternatives [13].

Denham et al. [4] characterized individual switching patterns using transition matrices [23], which contain the conditional probabilities for transitions between perceptual alternatives. This method gives a richer characterization of switching behaviour than that provided by the number of switches or percept proportions and phase durations alone, or even together. If required, all of these traditional measures can be extracted from the transition matrices. However, by pooling data firstly from the entire participant set to build a ‘global’ transition matrix, and then by participant and/or condition, the probabilities of any missing perceptual alternatives, or specific transitions, can be assigned in a principled and unbiased way (for more details, see [23]). This makes the transition matrices suitable for comparing individuals based on a multidimensional description of their switching patterns without the need for *ad hoc* probability estimates or for excluding participants who do not report all the perceptual alternatives. By comparing the Kullback-Leibler divergence (K-L distance, [24]) metric between transition matrices describing the same listener’s switching patterns in separate experimental sessions with those of different listeners, Denham et al. [4] found that switching patterns were significantly more similar within than between listeners even when the sessions were separated by more than a year. This suggests that individuals display idiosyncratic switching patterns, which are highly stable over time.

Here we asked what other characteristics of individuals may be related to their idiosyncratic perceptual behaviour displayed in the auditory streaming paradigm. Frontal-lobe functions are known to affect the perception of ambiguous stimuli (see, e.g., [25–27]).Because executive functions are usually linked to the frontal lobes [28–30], we decided to explore correlations between individual differences in three classes of executive functions [31] and switching patterns in the auditory streaming paradigm.

The first executive function class according to Miyake et al. [31] is termed *set shifting*, which refers to the ability to shift between tasks or sets of information required to solve that task. Developmental studies [32,33] showed no significant relation between this ability and individual differences in ambiguous figure reversals (switching rate). However, to the best of our knowledge, no adult studies have tested the possible role of set shifting in the perception of ambiguous stimuli.

The second class of executive functions [31] is *updating* or *working memory*, which refers to the capacity to hold on to relevant information. There is some controversy regarding whether working memory capacity and working memory updating represent similar or different functions. There is evidence that updating tasks also measure working memory capacity [34] as well as evidence discriminating the two [35]. It has been shown that larger working memory capacity was related to longer intervals with no change in perception (termed “perceptual phase”) in binocular rivalry [36] and reversible figures [37]. Further, individuals with higher verbal but not spatial working memory capacity were more successful in voluntarily biasing their perception of ambiguous figures [38].

The third class of executive functions [31] is *inhibition*, which encompasses three interrelated processes, namely interference control, aborting an ongoing response, and inhibition of a prepotent response [39]. Some neural models of multi-stable perception (e.g., [40,41]) suggest that inhibition and adaptation are two low-level processes that determine the dynamics of perception of ambiguous stimuli. Neural-level inhibition and inhibition as an executive function are not equivalent, thus they could affect perception differently. Developmental studies show that inhibition as an executive function is required for switching between possible interpretations of ambiguous stimuli [33]. In the current study, we assessed the relationship between these three classes of executive functions and the switching patterns recorded using the auditory streaming paradigm.

Inter-individual variation in personality traits may also be related to individual differences in perceptual switching. In addition to the neural-level inhibition and inhibition as an executive function described above, it is possible that a third type of inhibition, namely impulsivity—the lack of inhibition as a personality dimension could also be related to perceptual switching patterns. Only one previous study showed that impulsive individuals switched less than less impulsive ones, but this study measured impulsivity indirectly [42].

We also explore the effects of some general personality traits, such as the ones measured by the Five Factor Model (termed “extraversion”, “agreeableness”, “conscientiousness”, “emotional stability”, and “openness”[43]), on the perception of ambiguous stimuli. A classic study on the relationship between reversal rate and personality found that introverts switch less than extroverts [44]. Recently, Bosten et al. [45] found that indicators of perceptual behaviour are related to both extraversion and agreeableness in dichoptic masking, but not in binocular rivalry or stereo acuity.

Another possibly related personality meta-trait is ego-resiliency (ER), which assesses the adaptive flexibility of behaviour [46–48]. Individuals with high ego-resiliency are able to flexibly coordinate their behaviour with situational demands and behavioural possibilities in an adaptive way. They can also be described as having an open-minded experience and information-seeking tendency. Individuals with low ego-resiliency tend to perseverate rather than responding adaptively to environmental demands. Flexibility defined by ER could be a factor that is related to the individual differences observed in perceptual switching.

In addition, the Internal and External Encoding Styles Scale [49] was considered as a possible correlate of individual switching patterns in the auditory streaming paradigm. This is based on the notion that the amount of external information required to activate a pre-existing schema depends on the encoding style of the individual: the more internal one’s encoding style, the less information is required and vice versa. Further, the more internal one’s encoding style, the greater the probability that interpretation of external information will be based solely on pre-existing schemas. In contrast, the more external one’s encoding style, the more likely it is that the schema will be modified based on new information. In multi-stability paradigms the external information is constant, thus internal coding styles might play an important role in the variability between individuals.

Finally, higher creativity has been previously related to increased rates of switching between alternative interpretations of ambiguous figures [50,51]. There are several competing and complementary theories of creativity [52] and no consensual definition has yet emerged [53]. Creativity is typically assessed by measuring ideational fluency, uniqueness or originality, and flexibility of thinking [54]. The most commonly used measure is that of divergent thinking, the ability to generate new ideas from a wide range of cues, experiences, ideas, and information [55]. We included divergent thinking tests to assess creativity in our study.

In summary, the aim of the current study was to identify idiosyncratic switching patterns in the perception of auditory streaming and to explore their possible correlation with three types of psychological constructs: executive functions, personality traits, and creativity. Some of these constructs have been implicated in previous research as being related to the way individuals experience ambiguous stimuli, while other constructs included here have so far not been considered. The current study attempted to conduct an explorative, yet systematic investigation of possible relationships between these different constructs and individual differences in multi-stable perception.

## Methods

### Participants

Fifty-three healthy volunteers (41 female; 18–42 years, *M*_age_ = 22.38, *SD*_age_ = 4.04) participated in the experiment. All participants reported having normal hearing. None of the participants were taking any medication affecting the central nervous system. Participants gave written informed consent after the experimental procedures had been explained to them. The study was approved by the institutional review board of the Institute of Cognitive Neuroscience and Psychology, Research Centre for Natural Sciences of the Hungarian Academy of Sciences, Budapest, Hungary. Participants either received modest financial compensation or extra credits in a university course for their participation. One participant’s data was excluded from the main experiment, because she reported hearing only one perceptual state throughout all test blocks. Although this is a valid response, the participant is an extreme outlier within the group whose perceptual tendencies cannot be correctly represented in the statistical analyses. Four participants were excluded from the analysis based on poor catch-trial matching (see below) in the auditory streaming task. Thus, the sample analysed was based on forty-eight participants (37 female; 18–42 years; *M*_age_ = 22.46, *SD*_age_ = 4.19).

### Measures

#### Auditory perceptual task

Sinusoidal tones of 75 milliseconds (ms) duration (including 10 ms rise and fall times) with an intensity of 45 dB sensation level (hearing threshold measured individually for each participant with a staircase procedure using the experimental sounds) were presented according to the auditory streaming paradigm (a cyclically repeating”ABA-”pattern; Fig 1, top panel). The frequency difference was 4 semitones with the”A” tone frequency set at 400 Hz and”B” tone at 504 Hz. The stimulus onset asynchrony (SOA, onset to onset time interval) was 150 ms. Participants were presented with eleven four-minute-long sequences with an additional ca. 40 second catch-trial segment (see the Test procedure section) appended without a break to the end of each block. Tones were delivered through Sennheiser HD600 headphones by an IBM PC computer using the Cogent 2000 toolbox [56] under MATLAB [57].

Listeners were instructed to mark their perception continuously in terms of four possible categories: a) “integrated” (ABA-; Fig 1, second panel; response: depressing one of the two response keys), b) “segregated” (A-A-/B—; Fig 1, 3rd panel; depressing the other response key), c) “combined” (-AB-/—A or -BA-/A; Fig 1, bottom panel; simultaneously depressing both response keys), and d) “none” (no repeating pattern perceived; releasing both response keys). Participants received both instructions and training to help them identify the different perceptual categories. The description of the integrated percept emphasized hearing all tones as part of a single repeating pattern with a galloping rhythm. The description of the segregated percept emphasized hearing two isochronous sound streams in parallel, one in the foreground, the other in the background, each with a uniform (one slower, the other faster) delivery rate. The description of the combined percept emphasized the perception of two parallel streams of sound, at least one of which included a repeating pattern composed of both high and low tones. Finally, the “none” choice allowed listeners to indicate that they did not hear any repeating pattern or could not decide between the patterns previously described to them. The left and right arrow keys of a standard Hungarian IBM PC keyboard were used as response keys with their roles counterbalanced across participants. Participants were instructed to keep one or both keys depressed for as long as they continued hearing the corresponding pattern but to switch to another combination as soon as their perception changed. They were asked to employ a neutral listening mind-set (termed “neutral instructions”; for a detailed discussion of the instructions, see [13]). The state of the response keys was continuously recorded at a nominal sampling rate of 10Hz.

Participants were trained to indicate their perception in terms of the above listed four categories without hesitation. Training started with the participant listening to six one-minute long demonstration sequences, each promoting the perception of one of the alternatives shown in Fig 1. The integrated percept was introduced by using a smaller frequency difference between the “A” and “B” tones (1ST; 400 and 426 Hz, respectively) than the parameters chosen for the experiment, while the segregated percept was initially demonstrated by using a larger frequency difference (890 Hz). Subsequently, the two segregated and the three combined percepts in Fig 1. were demonstrated by emphasizing the corresponding repeating tone pattern within an auditory streaming sequence with the parameters used in the experiment. Emphasis was created by attenuating by 18 dB those tones which were not part of the intended foreground stream and their timbre was also manipulated by including eight harmonics with equal weights (thus promoting perception of the normal-intensity puretones as a single coherent sequence). After the response key assignment and the “none” choice were explained to the participant, training continued in blocks of six sequences separated by short silent intervals. The first sequence was one-minute-long and its parameters were identical to those used in the experiment. This was followed by five sequences of 6–9 s, each, one sequence for each of the categories the participant was required to identify. The order of the five short sequences (small-frequency-difference sample and the 4 attenuated-intensity tones examples), which served as catch trials, was randomized separately for each training block. The training was completed when the participant made the intended response for at least 80% of the presentation time during the catch trials or when 20 training blocks had been delivered. During the training blocks, the experimenter gave feedback and further help as needed. No participant was rejected on the basis of the accuracy of their responses within the training blocks.

A ca. 40-second long catch-trial period, consisting of the five 6–9 s long segments of the example patterns used in the demonstration blocks, was appended to the end of each four-minute stimulus block in the experiment, with the purpose of checking correct response assignment for the non-ambiguous patterns throughout the experiment. The order of the catch-trial patterns was randomized across participants and blocks with each pattern appearing only once within a catch-trial period. If the average of the catch-trial matching performance across perceptual patterns and blocks during the experiment was below 60%, the participant was excluded.

#### Executive functions

Inhibition, updating, and shifting [31] were assessed as follows. Inhibition was measured using a computerized version of the Stroop task [58,59] using E-Prime 2.0 [60]. The colours red, green, and blue were used, and participants were instructed to press the arrow key assigned to the colour appearing on a 15.6” screen with a resolution of 1366 x 768 pixels as quickly as possible. Participants were approximately 60cm away from the screen and stimuli had a width of 12cm and height of 3cm, giving them a vertical visual angle of 2.9° and horizontal visual angle of 11.4°. The arrow keys were used as response keys: “up” was paired with red, “left” was paired with blue, and “right” was paired with green. The task consisted of four conditions, each measured by 60 trials and delivered in three stimulus blocks. The first block was the neutral-word condition. In this condition, the names of the three colours appeared on the screen written in black. In the neutral-colour condition (2nd block) four “X” letters appeared in one of the three colours. The number of the “X” letters was decided by the average number of letters (4) in the Hungarian names of the colours used in the experiment. The third and final block mixed together the congruent and the incongruent condition in equal proportion. In congruent trials, the colour names appeared in their respective colours, whereas in the incongruent trials, the colour names appeared in one of the other two colours. The participants’ task was to press the correct response key as quickly and accurately as they could. The stimuli were shown on the screen until one of the response keys was depressed. This was followed by a blank screen for 250 ms. The Stroop interference effect was measured in the following way: first, the median reaction times of the correct responses measured in the colour-neutral and word-neutral conditions were averaged to get a neutral reaction time; second, the neutral reaction time was subtracted from the median reaction time of the correct responses in the incongruent conditions. Thus, a smaller reaction time difference indicates stronger inhibitory control of a prepotent response. The task was run using E-prime 2.0.

The executive function of updating was measured using a computerized version of the N-back task [61] using PsychoPy2 [62]. The same screen and viewing distance was used as in the Stroop task (see above). Stimuli had a width and height of 5cm each, giving visual angles of 4.8°. The task consisted of three blocks, each with 50 trials, of which 20 trials contained targets. 14 different capital letters were used as stimuli. Each letter was presented for 500 ms followed by a blank screen for 250 ms. In the first stimulus block, the participant’s task was to press a key when the current stimulus matched the preceding one (1-back condition). In the second and the third stimulus block, the letters to be matched were separated by one (2-back condition) and two letters (3-back condition), respectively. Responses were recorded during the 500 ms intervals of letter presentation but not during the 250-ms blank intervals. A response that was made within 500 ms from the onset of a target letter was scored as a hit, whereas a response within that time frame for a non-target was coded as a false alarm. Corrected Recognition Rate (CRR) was calculated for each condition using the following formula: (Hits / Targets)–(False alarms / Non-targets). The 1-back condition showed a ceiling effect, thus only the CRRs from the 2- and 3- back conditions were taken into account for further analysis.

Shifting was measured by semantic fluency [63], where participants were asked to name as many animals as they could. Unknown to them, they had one minute to complete the task. Responses were written down by the experimenter. During the spontaneous production of words, participants often name animals from various subcategories, such as African animals or pets. Shifts between these subcategories or semantic clusters can be used to assess the shifting executive function. Identification and analysis of the clusters was based on the protocol of Troyer and colleagues [63,64], adapted to Hungarian by Mészáros, Kónya, and Csépe [65] and was scored by an independent rater. Cluster-size was given as the length of the cluster minus one, such that a single word from a category was regarded as a cluster of the size zero, while for example, a cluster with five consecutive words belonging to the same category had the size four. The average cluster size and the number of subcategory changes (number of clusters minus one) were used as the output measures. All participants had at least 18 correct responses.

#### Personality questionnaires

Participants completed four personality questionnaires on a computer using E-prime 2.0 software [60]. Computer-based administration helped to ensure that all participants responded to all questions, thus, there were no missing values in the final dataset.

First, they completed the ER89 questionnaire [66], which measures ego-resiliency (ER). The ER89 is a 14-item questionnaire, where participants have to indicate to what extent the items apply to them on a four-level Likert scale from “Does not apply to me at all” to “Applies to me very much”. In the Hungarian version [48], ER is measured using eleven items of the questionnaire (Cronbach’s α = .639 for the current sample).

Participants then filled out the Big Five Inventory [67] (Hungarian adaptation by personal communication from Z. Vass, Károli Gáspár University of the Reformed Church in Hungary), which measures the big five traits: Extraversion (8 items, α = .768 for the current sample), Agreeableness (nine items, α = .740), Conscientiousness (9 items, α = .833), Emotional stability (8 items, α = .855), and Openness (10 items, α = .806). Participants were instructed to rate the items on a 5-level Likert scale ranging from”I strongly disagree” to “I strongly agree”.

The next inventory was the UPPS Impulsive Behavior Inventory (Hungarian adaptation by personal communication from G. Orosz, Hungarian Academy of Sciences [68]). This inventory consists of 45 items that assess the four different aspects of impulsivity, labelled Urgency (12 items, α = .850 for the current sample), (lack of) Premeditation (11 items, α = .881), (lack of) Perseverance (10 items, α = .862), and sensation seeking (12 items, α = .881). Items on the questionnaire are scored on a 4-level Likert scale from ‘‘I strongly agree” to ‘‘I strongly disagree”.

Next, the Internal/External Encoding Style Questionnaire (NISROE [49]) was completed (the Hungarian version was adapted by the authors following the protocol of [69]). NISROE measures the internal and external encoding styles on 21 items using a 6-level Likert scale ranging from”I strongly disagree” to “I strongly agree”. As suggested by Lewiczki [49], only items 5, 8, 11, 15, 18, and 21 are used to calculate the final score on the questionnaire (α = .708 for the current sample).

Finally, participants filled out the Balanced Inventory of Desirable Responding (BIDR; [70,71] Hungarian adaptation by personal communication from G. Orosz, Hungarian Academy of Sciences). This questionnaire uses 20 items to measure two dimensions, self-deceptive positivity (SDP, α = .790) and impression management (IM, α = .562), to assess individual sensitivity to social expectations. Respondents indicate their agreement with items on a seven-level Likert scale ranging from “Not true” to “Very true”. The scores of this questionnaire can be used to identify respondents whose responses are likely to be affected by implicit expectations present in the social context. We have reported in a separate paper that the social desirability bias as measured by the BIDR did not have a significant effect on perception [72] in the present dataset. Therefore, the data obtained with this instrument will not be reported here.

#### Creativity

Creativity was measured through two divergent thinking tasks, because this aspect of creativity can be best assessed in laboratory settings [73]. The first task was the Use of Objects Task [74], in which participants were instructed to produce as many novel uses as they could for three common objects (brick, paperclip, and newspaper). They had one minute for each word. Their voice was recorded and later transcribed. The second task was caption generation [73] in which participants were instructed to write as many captions as they could for three “New Yorker Magazine” cartoons, which were provided to the authors by R. E. Jung (University of New Mexico) through personal communication. Participants had two minutes for each of the cartoons and recorded the captions using paper and pen. All participants produced at least one response for each task.

Three independent raters (2 females) with MA in psychology (age: 25–28 years; M = 26.33, SD = 1.53) assessed the responses using the consensual assessment technique [75–77]. They rated 283 captions and 1106 uses of common objects in a randomized order on a 6-level Likert scale ranging from “Not creative at all” to “Very creative”. Raters were instructed to attempt to achieve a normal distribution of the assigned levels and creativity was not defined to them. Inter-rater reliability was assessed as adjusted inter-class correlations with the Reliability Calculator 1.5 (Mangold International GmBH). Inter-rater reliability was .811 for the Use of Objects and .706 for the caption generation task, both being acceptable values. Item ratings were averaged and linearly transformed to the 0–1 range, separately for the two tasks. The average of the two task-ratings were taken into account as the Composite Creativity Index (CCI, inter-rater reliability = .797) of each participant.

### Procedure

The experimental session started with the questionnaires followed by the auditory streaming sequences and ended with the executive function and creativity tasks. The order of the questionnaires was fixed, whereas the tasks within the executive functions and creativity categories were counterbalanced across participants. The auditory streaming segment consisted of the training part followed by 11 experimental blocks. During the first five blocks, participants reported their perception according to the neutral instructions described above. For three of the remaining blocks, participants were instructed to hold on to each percept as long as they could (Hold condition) while still reporting their perception faithfully. For the remaining three blocks, participants were instructed to switch to another percept as soon as they could (Switch condition) while also marking their perception truthfully. The order of these two biased conditions was counterbalanced across participants. Data obtained in the two biased instruction conditions have been reported separately [72]. Only the data recorded using the neutral instructions are reported here. Breaks were included when switching tasks and between blocks as needed. The session, including questionnaires, lasted altogether for ca. 180 minutes.

### Data analysis

Variables were grouped into four categories based on the different aspects of cognition they represent: perceptual variables (8), personality or questionnaire-based variables (11), executive function variables (5), and creativity variables (3). Normality was assessed using the skewness and kurtosis of each variable distribution. Reliability of the questionnaires was assessed using Cronbach’s alpha. Data were analysed using MATLAB 2014a [57] and R 3.2.1. [78].

#### Pre-processing perceptual data from the auditory streaming paradigm

Perceptual phases shorter than 300ms were discarded from the data recorded in the auditory streaming paradigm, because these were assumed to result from inaccurate switching between key combinations (see [79]). The data removed by this pre-processing step amounted to 0.5% of the total record duration.

Transition matrices were constructed from the perceptual reports using the method described in [4,23]. Each transition matrix had 4 rows and 4 columns (each corresponding to one of the 4 alternative perceptual classes, described previously) with elements representing the conditional probability of the percept changing from the one assigned to the column (starting percept) to the one assigned to the row (next percept; including the probability of no percept change). The conditional probabilities were estimated for each listener and block separately (block transition matrix), but also for each listener (by pooling data from all blocks for each listener: listener transition matrix), and for the whole experiment (by pooling all data from all neutral blocks for all participants: global transition matrix). Denham et al. [23] showed that the global transition matrix can be used to estimate missing data for individual listeners (i.e., transitions that were not observed for a given listener) and listener transition matrices can be used to estimate missing data for the each individual's block transition matrices. Therefore, this procedure was employed to provide a principled method for assigning default values in the event of missing data. Because the switching patterns obtained in the very first experimental block substantially differed from those obtained in the subsequent blocks [72], data from the first block were excluded. Four participants did not experience the combined percept. In the whole sample 37.67% of the sixteen possible transitions were missing. Most of the missing transitions were related to the ‘none’ percept, whose overall proportion was less than 0.8% in the data. Since we did not analyse the ‘none’ responses, the effective proportion of missing transitions was 13.19%.

#### Perceptual variables

Most previous studies investigating individual differences in perceptual multi-stability (e.g., [21]) measured perceptual variables, such as the proportions and average phase durations of the alternative perceptual reports as well as the number of switches between the alternative perceptual reports. In order to allow comparisons between the current and previous results, the mean number of switches, and the proportions and mean phase durations of the integrated, segregated, and combined perceptual reports were estimated from the listener transition matrices. Variables for the “none” perceptual report were not analysed, because the overall proportion of this percept was less than 0.8%. The remaining variables characterizing the perception of the tone sequences were used in the analyses to be reported (see below).

Motivated by the idea that some listeners might discover the perceptual alternatives faster than others, we also analysed the time to discover all three perceptual alternatives, which is a potential source of individual differences in the data. This new measure provides information about the viability of the possible perceptions. Short discovery times suggest that all alternatives are relatively easy to perceive for the given participant, whereas long discovery times suggest that some perceptual alternatives are less viable; i.e. more difficult to discover and/or less able to win the competition for perceptual dominance. The measure was calculated by means of simulation: the listener transition matrices were used to simulate switching behaviour. Each simulation was run until all patterns were discovered. For each listener, the median duration of 1000 simulation runs was used as the “Time to discover all patterns” value. Lower values indicate quicker discovery of all perceptual patterns. The descriptive statistics of all variables are included in the S1 Table.

#### Testing the presence of idiosyncratic switching patterns

In order to determine whether a given listener showed an idiosyncratic switching pattern, the intra-individual and inter-individual similarities were calculated for each participant and compared using Wilcoxon’s Signed Rank test. Intra-individual similarity was assessed by calculating the K-L distance values between each pair of the 4 (2nd to 5th) block transition matrices of the same listener. Inter-individual similarity was assessed by calculating the K-L distance values between transition matrix pairs for each combination having one of the 4 blocks of one listener and one of the 4 blocks of every other listener. In the Wilcoxon’s Signed Rank test, one-tailed assessment of significance was employed, because intra-individual similarity was expected to be lower than inter-individual similarity if the listener showed an idiosyncratic switching pattern. In previous studies (e.g. [3]), inter-individual variability was investigated using the number of switches measure. Therefore, we repeated the above analysis using the number of switches. The possibility of a different number of listeners being identified as having an idiosyncratic switching pattern by the two methods was tested with McNemar’s test [80].

#### Multidimensional Scaling of the transition matrices

Based on the K-L dFistances between the listener transition matrices, Multi-Dimensional Scaling (MDS [81]) was used to find the main dimensions characterising listeners’ perceptual switching behaviour in the auditory streaming paradigm. MDS is similar to factor analysis, but instead of using the covariance matrix of the investigated variables, it uses a distance measure between the points in order to extract the underlying dimensions. The scree test [82], which evaluates the stress values of the dimension configurations was used to decide on the number of dimensions to be used. The stress value assesses how well the observed distance matrix is reproduced by an MDS configuration. A linear stress criterion was used as an index of the goodness-of-fit, which is the stress normalized by the sum of squares of the inter-response distances. The scree test indicated that three dimensions were sufficient for describing the transition matrix space of the listeners' switching patterns (MDS stress = .0408).

Interpretation of the MDS dimensions was attempted by correlating them with the perceptual variables. Because some of the variables did not have a normal distribution, and a normality transformation could have distorted their magnitude, Spearman’s rank-order correlations were used for this analysis. The significance levels of the correlations were determined by two randomization (permutation) methods. In the first approach, the distribution of the correlation coefficients under the null hypothesis was estimated for each perceptual variable separately, by permuting the values of the given perceptual variable and correlating them with the given MDS dimension 10,000 times. The *p*-value of the observed correlation was established as the proportion of random correlations higher than or equal to the observed value (more precisely, the absolute values of the correlation coefficients were compared to obtain a two-tailed test). The second method controlled for the family-wise error rate of the above procedure by registering the highest (absolute) correlation between the given MDS dimension and all perceptual variables in each permutation run, and using the distribution of these maximal coefficients to compute the *p*-value of the observed correlations [83,84].

#### Correlations between perceptual and other variables

Spearman’s correlations were used to test the relationship between the MDS dimensions and personality, executive functions, and creativity variables. Correlations between these variables and the perceptual variables were also tested. Family-wise error correction was done as described in the section titled ‘Multidimensional Scaling of the transition matrices’, separately for the four groups of variables described at the beginning of the Data Analysis section. For correlations with significant *p*-values after family-wise error correction (marked as *p*_fwe_), both types of *p*-values are reported. The correlation matrix between the perceptual variables and the executive functions, personality traits, and creativity is included in S2 Table with *p*-values without family-wise error correction.

## Results

### Idiosyncratic Switching Patterns

42 of 48 participants (87.5%) showed significantly higher intra-individual similarity than inter-individual similarity, and hence an idiosyncratic switching pattern, as assessed by transition matrix distances. Using the number of switches to measure individual similarity, the number of listeners showing idiosyncracity was numerically, but not significantly (McNemar’s test: *p* = .424) lower: 38 of 48 participants’ number of switches (79.17%) was distinguishable from that of the rest of the participants (for individual results with Wilcoxon *Z* values see Table 1).

**Table 1:** Participants with idiosyncratic switching patterns based on transition matrices (Trans. Mat.) and number of perceptual switches (# of Switches) by Wilcoxon's Z values.

Participant	Trans. Mat.	# of Switches	Participant	Trans. Mat.	# of Switches
1	-3.174**	-2.702**	25	-3.239**	-1.981*
2	-3.505***	-2.281*	26	-1.933*	-1.381
3	-3.413***	-1.439	27	-3.222**	-1.822 *
4	-1.875*	-1.914*	28	-3.208**	-1.961*
5	-3.966***	-0.484	29	-1.635	-4.035***
6	0.257	-0.207	30	-0.171	-3.045**
7	-2.580**	-1.038	31	-3.295***	-2.955**
8	-3.939***	-2.446**	32	-3.908***	-2.590**
9	-2.470**	-1.594	33	-2.841**	-1.796*
10	-1.089	-1.849*	34	-2.726**	-1.154
11	-4.158***	-3.211**	35	-2.416**	-1.704*
12	-2.477**	-3.627***	36	-2.653**	-0.508
13	-3.724***	-3.507***	37	-1.753*	-2.386**
14	-4.222***	-1.212	38	-2.758**	-2.663**
15	-3.775***	-2.504**	39	-3.333***	-3.786***
16	-3.954***	-1.865*	40	-0.407	-1.703*
17	-1.665*	-2.217*	41	-3.550***	-2.187**
18	-3.522***	-2.631**	42	-3.007**	-2.622***
19	-2.206*	-2.562**	43	-0.392	-1.691*
20	-4.222***	-1.813*	44	-3.778***	-1.678*
21	-3.266**	-1.341	45	-3.846***	-2.477**
22	-3.732***	-3.391***	46	-1.717*	-2.528**
23	-2.367**	-1.892*	47	-4.220***	-1.719*
24	-1.998*	-3.062**	48	-3.346***	-2.934**

Participant = participant number, Trans. Mat. = Wilcoxon’s Signed Rank test *Z*-values from the transition matrix based testing, # of Switches = Wilcoxon’s Signed Rank test *Z*-values number of switches based testing

*** *p* < .001

** *p* < .01

* *p* < .05

### Correlates of the Perceptual Variables

High scores on the Stroop task (indicating less inhibition) were negatively related to the duration of the segregated phases (*r*(48) = -.370, *p* = .012, *p*_fwe_ = .049) and positively to the mean number of switches (*r*(48) = .372, *p* = .008, *p*_fwe_ = .036). The average cluster size in the fluency task as a measure of set shifting was positively related to the proportion of the integrated perceptual reports (*r*(48) = .374, *p* = .009, *p*_fwe_ = .043). This suggests that the ability to access a higher number of elements within a cluster in one run during the fluency task correlates with increased experience of the integrated perceptual state.

Of the personality dimensions, Ego-resiliency (*r*(48) = .443, *p* = .002, *p*_fwe_ = .018) was positively related to the proportion of the combined percept. Thus, ego-resilient individuals experience more combined percepts than ego-brittle ones. None of the other personality scores nor any of the creativity measures were significantly linked to any of the perceptual variables. For the other correlation coefficients, see S2 Table.

### Multi-Dimensional Scaling and Correlations with the MDS Dimensions

**Fig 2:**
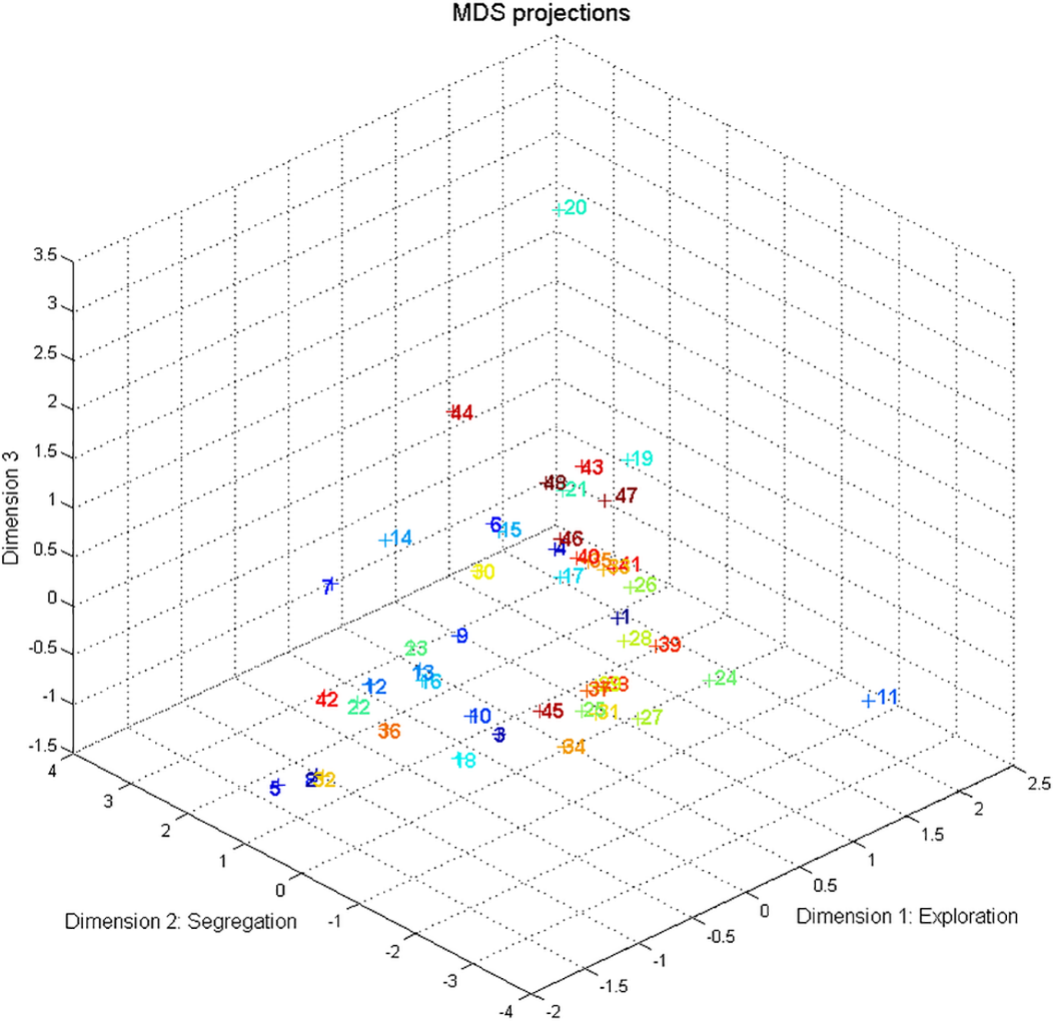
The Kullback-Leibler distances between listener transition matrices visualized on the dimensions extracted by Multi-Dimensional Scaling. Coloured numbers mark the participants.

The first MDS dimension was related to all perceptual variables with the exception of the proportion of segregated (Fig 2. and Table 2). Its most prominent correlates are the proportion of combined (*r*(48) = .998, *p* < .001, *p*_fwe_ < .001), the proportion of integrated (*r*(48) = -.800, *p* < .001, *p*_fwe_ < .001), the time to discover all patterns (*r*(48) = -.568, *p* < .001, *p*_fwe_ < .001), and the number of switches (r(48) = .534, p < .001, *p*_fwe_ = .001). Based on these correlations we term this dimension “Exploration”, because participants who switched more, required less time to discover all patterns, reported more combined (the least common perceptual organization, *M* = 19%, *SD* = 16%) and fewer integrated experiences (the most frequently experienced perceptual organization, *M* = 45%, *SD* = 15%) are placed at one end of the range in this dimension. At the other end of the range are the participants who switched less, required more time to discover all perceptual patterns, and experienced more integrated but fewer combined perceptual reports. The second dimension was linked only to the proportions of the segregated (*r*(48) = -.899, *p* < .001, *p*_fwe_ < .001) and the integrated perceptual reports (*r*(48) = .571, *p* < .001, *p*_fwe_ < .001). Based on these correlations, we term this dimension “Segregation”, because it separates the participants who experienced more integrated and less segregated percepts from those showing the opposite pattern. The third dimension was not linked significantly to any of the perceptual variables.

**Table 2:** Spearman’s Rank Order correlation coefficients between the Multi-Dimensional Scaling dimensions and the perceptual variables.

	MDS X	MDS Y	MDS Z
Proportion of integrated	-.800***	.571***	.035
Proportion of segregated	-.242	-.899***	.024
Proportion of combined	.998***	-.057	-.086
Duration of integrated	-.436*	.074	.103
Duration of segregated	-.428*	-.309	.211
Duration of combined	.282	.047	.222
Number of switches	.534**	-.056	-.102
Time to discover all	-.568***	.291	.179

MDS X = the first dimension of the MDS, MDS Y = the second dimension of the MDS, MDS Z = the third dimension of the MDS, Duration of integrated/segregated/combined = average phase duration of the integrated/segregated/combined perceptual reports, Number of switches = average number of switches, Time to discover all = time to discover all patterns.

*** *p*_fwe_ < .001

** *p*_fwe_ < .01

* *p*_fwe_ < .05

Ego-resiliency (*r*(48) = .444, *p* = .002, *p*_fwe_ = .020) was positively related to the Exploration dimension. These results suggest that individuals scoring higher on Ego-resiliency are likely to be more explorative than those who score lower on this meta-trait. It appears that this explorative tendency is one of the main sources of the explained variance between the participants in this multi-stable perceptual situation. No other personality trait, executive function, or creativity measure was significantly related to any of the MDS dimensions. All correlation coefficients between the perceptual variables and the executive functions, personality traits, and creativity can be found in the table of S2 Table.

## Discussion

The aim of this study was to explore the executive function, personality trait, and creativity-related correlates of inter-individual variability of switching patterns in multi-stable auditory perception. Most participants displayed an idiosyncratic perceptual switching pattern both in terms of the number of switches and in their patterns of switching, as characterized by the transition matrices. Thus, the finding of Denham et al. [4] who studied six participants in numerous sessions, was replicated for a larger sample and with only four perceptual alternatives (as opposed to the 7 alternatives used in [4]). Numerically, a higher number of participants were identified as having an idiosyncratic perceptual switching pattern using the transition matrix description than using the number of switches, although the difference was not significant. Thus the number of switches proved to be a useful indicator of individual differences even though the transition matrix contains additional information about the dynamics of perceptual switching.

The main dimensions on which listeners’ switching patterns differed from each other were identified as Exploration (positively correlated with the proportion of combined, and the number of switches, whereas negatively correlated with the proportion of integrated and the time to discover all patterns) and Segregation (positively correlated with the proportion of the integrated perceptual reports and negatively with that of the segregated perceptual reports). We found that only a personality meta-trait termed Ego-resiliency [46] (ER) was significantly related to the Exploration dimension. Further, ER was also significantly related to the proportion of the combined percept, which is the least common perceptual organization experienced in the current auditory streaming paradigm and which is also the most prominent perceptual correlate of the Exploration MDS dimension. Exploration can be defined as *“any behaviour or cognition motivated by the incentive reward value of uncertainty”* ([85], p. 2) and it is important to note that *“the brain addresses these questions both consciously and unconsciously”* ([85], p. 2). This measure can indicate an unconscious tendency to explore as much of the environment as possible to reduce uncertainty. However, a conscious, top-down type of exploration is also possible during the task. It is not possible to decide between these two alternatives based on the present data, but it seems that the flexibility and open-minded experience-seeking tendency of the individual measured with ER is related to this explorative tendency during the auditory streaming task. No other personality, executive-function, or creativity measure used in the current study was significantly correlated with this or the other two MDS dimensions.

Two executive functions, inhibition and shifting, were significantly related to individual differences in perceptual variables. Higher inhibition levels were related to longer average segregated phase durations and to a lower number of switches. Developmental findings reported that more inhibition is required to be able to switch [33]. Children start to switch between possible interpretations of multi-stable stimuli at about the age of 5, as they are not able to switch before that. Frontal functions are required for switching (e.g. [25]), and frontal brain areas are also developing during this period and up to puberty [86]. Thus, it is possible that the positive relationship between the number of switches and inhibition observed in developmental studies represent the parallel maturation of these functions, thus the relationship is due to a shared source. It should be noted that there are large differences between the current and the referenced developmental studies both in the stimuli and the instructions used. The significant relationship found between the duration of the segregated phases and inhibition (as measured by the Stroop task) is compatible with models of auditory stream segregation based on competition between alternative groupings of sound (proto-objects; e.g. [87]), as in these models proto-objects mutually inhibit each other. However, the actual mechanisms by which inhibition as an executive function affects auditory stream segregation are not yet known.

Shifting was significantly related to the proportion of integrated perceptual reports, with higher shifting values corresponding to lower proportions of integrated perceptual reports. In a developmental study of multi-stable perception, Wimmer and Doherty [33] found that shifting is not required for the development of the ability to switch between alternative interpretations of an ambiguous stimulus. Thus, perhaps, shifting is related to fine-tuning the individual switching patterns, rather than being a necessary prerequisite of switching. Again, however, one should note that Wimmer and Doherty [33] used a different measure of shifting, and different stimuli and instructions for this measurement, which may also explain the differences in the results. Finally, as for the third main executive function, previous studies indicated a link between working memory capacity and inter-individual variability in perceptual multi-stability [36,37]. The current results did not provide corroborative evidence for these findings. Although some studies showed that working memory capacity tasks may also measure updating and *vice versa* [34], other evidence suggests that the two are different constructs [35]. Future research is needed to investigate whether the working memory capacity and working memory updating have different effects on individual differences in different forms of perceptual multistability.

Neural models of perceptual multi-stability are generally based on the assumption that there are three effects responsible for switching between alternative interpretations [40,41,88]. The first one is adaptation, which refers to the characteristic that the perceptual system gradually adapts to the dominant interpretation; thus explaining the inevitability of switching. The second one is inhibition, which underlies the competition for dominance by inhibiting the currently non-dominant perceptual alternatives. Thus, this mechanism promotes stability by helping to maintain the current perceptual organization. The third factor is noise, which is responsible for the non-periodic, stochastic dynamics of switching behaviour. So, increasing inhibition leads to fewer switches and longer phase durations. Kondo et al. [89] found that the higher the concentration of the GABA inhibitory neurotransmitter in the auditory cortex, the fewer the switches and longer the phase durations experienced by participants in the auditory streaming paradigm. We observed two similar effects for the shifting and the inhibition executive functions: more shifting was related to lower proportions of the integrated percept, the percept that is typically dominant for a long period of time at the beginning of the stimulus block [13], whereas higher inhibition was related to lower switching rates (longer phase durations). Although shifting and inhibition as executive functions are different from neural-level adaptation and inhibition, they may represent top-down modulators of these low-level mechanisms.

In summary, the current study identified idiosyncratic switching patterns in an auditory multi-stability paradigm. We found that the dimension on which individuals primarily differed from each other was related to the number of switches, time required to discover all possible perceptual patterns, and the relative proportions of the least and most common percepts. This dimension of inter-individual differences was termed Exploration. It was significantly associated with Ego-resiliency, a meta-trait describing adaptive flexibility to meet the challenges of the environment. Individuals with high ego-resiliency were more explorative on the Exploration dimension than individuals with lower ego-resiliency. Inter-individual differences in the inhibition and shifting executive functions were found to be related to some indicators of perceptual switching. We assume that these executive functions may modulate the neural-level processes of inhibition and adaptation, which have been suggested as two important factors governing multi-stable perception. Future research may help to clarify commonalities and differences between the various multi-stable perceptual phenomena (i.e., modality, level of representation) and how the various individual capabilities (including working memory capacity) and personality traits interact in their effect on them.

## Supporting Information

S1 Table Descriptive statistics of the measured variables. Mean (SD) = the mean and the standard deviation of the variable, Min = the minimum of the variable, Max = the maximum of the variable, α = Cronbach’s alpha in the case of the personality questionnaires and inter-rater reliability in case of Creativity tasks, MDS X = the first dimension of the MDS, MDS Y = the second dimension of the MDS, MDS Z = the third dimension of the MDS, Duration of integrated = average phase duration of the integrated percept in seconds, Duration of segregated = average phase duration of the segregated percept in seconds, Duration of combined = average phase duration of the combined percept in seconds, Number of switches = average number of switches, Time to discover all = time to discover all patterns (in seconds), Stroop RT = median reaction time on the Stroop task in seconds, 2-Back CRR = Corrected Recognition Rate on the 2-back condition of the N-back task, 3-Back CRR = Corrected Recognition Rate on the 3-back condition of the N-back task, Fluency cluster size = average cluster size in the semantic fluency task, Fluency number of switches = average number of switches in the semantic fluency task, CCI = Composite Creativity Index.
(DOCX)

S2 Table Spearman Rank Order correlation coefficients between the perceptual and the executive functions, personality traits, and creativity. MDS X = the first dimension of the MDS, MDS Y = the second dimension of the MDS, MDS Z = the third dimension of the MDS, Duration of integrated = average phase duration of the integrated percept in seconds, Duration of segregated = average phase duration of the segregated percept in seconds, Duration of combined = average phase duration of the combined percept in seconds, Number of switches = average number of switches, Time to discover all = time to discover all patterns (in seconds), Stroop RT = median reaction time on the Stroop task in seconds, 2-Back CRR = Corrected Recognition Rate on the 2-back condition of the N-back task, 3-Back CRR = Corrected Recognition Rate on the 3-back condition of the N-back task, Fluency cluster size = average cluster size in the semantic fluency task, Fluency number of switches = average number of switches in the semantic fluency task, CCI = Composite Creativity Index. *** *p* < .001, ** *p* < .01 * *p* < .05
(DOCX)
