# Supplementary material for: Auditory Multi-Stability: Idiosyncratic Perceptual Switching Patterns, Executive Functions and Personality Traits
Source: PLoS One. 2016 May 2;11(5):e0154810. doi: 10.1371/journal.pone.0154810 (PMC4852918; doi:10.1371/journal.pone.0154810)
Supplement: S1 Table — Mean (SD) = the mean and the standard deviation of the variable, Min = the minimum of the variable, Max = the maximum of the variable, α = Cronbach’s alpha in the case of the personality questionnaires and inter-rater reliability in case of Creativity tasks, MDS X = the first dimension of the MDS, MDS Y = the second dimension of the MDS, MDS Z = the third dimension of the MDS, Duration of integrated = average phase duration of the integrated percept in seconds, Duration of segregated = average phase duration of the segregated percept in seconds, Duration of combined = average phase duration of the combined percept in seconds, Number of switches = average number of switches, Time to discover all = time to discover all patterns (in seconds), Stroop RT = median reaction time on the Stroop task in seconds, 2-Back CRR = Corrected Recognition Rate on the 2-back condition of the N-back task, 3-Back CRR = Corrected Recognition Rate on the 3-back condition of the N-back task, Fluency cluster size = average cluster size in the semantic fluency task, Fluency number of switches = average number of switches in the semantic fluency task, CCI = Composite Creativity Index. (DOCX) [file pone.0154810.s001.docx]

|  | Mean (SD) | Min | Max | Skewness | Kurtosis | α |
| --- | --- | --- | --- | --- | --- | --- |
| MDS dimensions |  |  |  |  |  |  |
| MDS X | 0.67 (1.05) | -1.79 | 2.05 | -0.38 | -0.73 | ― |
| MDS Y | 0.05 (0.53) | -3.15 | 3.90 | 0.84 | 5.81 | ― |
| MDS Z | -0.07 (0.33) | -1.17 | 3.26 | 1.22 | 1.29 | ― |
| Perceptual variables |  |  |  |  |  |  |
| Proportion of integrated | 0.45 (.15) | 0.01 | 0.80 | -0.11 | 0.24 | ― |
| Proportion of segregated | 0.35 (.12) | 0.00 | 0.56 | -0.86 | 1.84 | ― |
| Proportion of combined | 0.19 (.16) | 0.00 | 0.72 | 0.71 | 0.54 | ― |
| Duration of integrated | 11.81 (8.23) | 3.72 | 44.66 | 2.01 | 5.15 | ― |
| Duration of segregated | 8.22 (6.34) | 1.08 | 37.07 | 2.71 | 9.05 | ― |
| Duration of combined | 5.38 (3.91) | 0.34 | 18.60 | 1.41 | 2.23 | ― |
| Number of switches | 36.04 (16.18) | 9.76 | 66.27 | 0.34 | -0.91 | ― |
| Time to discover all | 135.27 (171.03) | 11.52 | 480.00 | 1.25 | -0.09 | ― |
| Executive functions |  |  |  |  |  |  |
| Stroop RT | 0.18 (0.13) | 29.00 | 699.50 | 1.98 | 4.89 | ― |
| 2-back CRR | 0.65 (0.21) | 0.05 | 1.00 | -0.78 | 3.18 | ― |
| 3-back CRR | 0.34 (0.20) | 0.05 | 1.00 | 1.25 | 5.04 | ― |
| Fluency cluster size | 1.40 (0.58) | 0.41 | 3.00 | 0.72 | 0.50 | ― |
| Fluency number of switches | 12.85 (4.27) | 6.00 | 27.00 | 1.07 | 1.54 | ― |
| Personality traits |  |  |  |  |  |  |
| Ego-resiliency | 32.08 (4.40) | 21.00 | 41.00 | -0.23 | -0.03 | .639 |
| Extraversion | 27.83 (5.41) | 17.00 | 37.00 | -0.22 | -0.85 | .768 |
| Agreeableness | 31.25 (5.58) | 13.00 | 40.00 | -0.94 | 1.46 | .740 |
| Conscientiousness | 31.83 (6.25) | 17.00 | 44.00 | -0.23 | -0.55 | .833 |
| Emotional stability | 23.46 (6.28 | 11.00 | 38.00 | 0.50 | 0.07 | .855 |
| Openness | 37.69 (5.72) | 18.00 | 50.00 | -0.83 | 1.81 | .806 |
| (lack of) Premeditation | 21.33 (5.86) | 12.00 | 42.00 | 0.62 | 1.82 | .881 |
| Urgency | 27.46 (6.91) | 15.00 | 45.00 | 0.23 | -0.10 | .850 |
| Sensation seeking | 32.13 (7.98) | 17.00 | 46.00 | -0.20 | -1.00 | .881 |
| (lack of) Perseverance | 18.98 (5.48) | 11.00 | 34.00 | 0.77 | 0.35 | .862 |
| NISROE | 21.21 (5.38) | 8.00 | 31.00 | -0.55 | -0.02 | .807 |
| Creativity |  |  |  |  |  |  |
| CCI | .57 (0.17) | 0.05 | 0.90 | -0.68 | 0.66 | .797 |
| Use of Objects task | .51 (0.18) | 0.00 | 0.93 | -0.27 | 0.86 | .811 |
| Caption generation task | 0.64 (0.24) | 0.00 | 1.00 | -0.65 | -0.16 | .706 |
