# Supplementary material for: Auditory Multi-Stability: Idiosyncratic Perceptual Switching Patterns, Executive Functions and Personality Traits
Source: PLoS One. 2016 May 2;11(5):e0154810. doi: 10.1371/journal.pone.0154810 (PMC4852918; doi:10.1371/journal.pone.0154810)
Supplement: S2 Table — MDS X = the first dimension of the MDS, MDS Y = the second dimension of the MDS, MDS Z = the third dimension of the MDS, Duration of integrated = average phase duration of the integrated percept in seconds, Duration of segregated = average phase duration of the segregated percept in seconds, Duration of combined = average phase duration of the combined percept in seconds, Number of switches = average number of switches, Time to discover all = time to discover all patterns (in seconds), Stroop RT = median reaction time on the Stroop task in seconds, 2-Back CRR = Corrected Recognition Rate on the 2-back condition of the N-back task, 3-Back CRR = Corrected Recognition Rate on the 3-back condition of the N-back task, Fluency cluster size = average cluster size in the semantic fluency task, Fluency number of switches = average number of switches in the semantic fluency task, CCI = Composite Creativity Index. *** p < .001, ** p < .01 * p < .05 (DOCX) [file pone.0154810.s002.docx]

|  | MDS X | MDS Y | MDS Z | Proportion of integrated | Proportion of segregated | Proportion of combined | Duration of integrated | Duration of segregated | Duration of combined | Number of Switches | Time to discover all |
| --- | --- | --- | --- | --- | --- | --- | --- | --- | --- | --- | --- |
| Executive functions |  |  |  |  |  |  |  |  |  |  |  |
| Stroop RT | .308* | .108 | -.195 | -.159 | -.161 | .298* | -.236 | -.370* | -.230 | .378** | -.159 |
| 2-back CRR | -.191 | -.115 | .041 | .127 | .192 | -.194 | -.058 | .071 | -.224 | -.029 | -.013 |
| 3-back CRR | -.093 | -.095 | .033 | -.007 | .195 | -.089 | .007 | .004 | -.146 | -.120 | .165 |
| Fluency cluster size | -.318* | .184 | .014 | .374** | -.075 | -.311* | .313* | .177 | .076 | -.277 | .322* |
| Fluency number of switches | .221 | -.135 | -.075 | -.262 | .053 | .213 | -.298* | -.148 | -.032 | .226 | -.362* |
| Personality traits |  |  |  |  |  |  |  |  |  |  |  |
| Ego-resiliency | .444** | .199 | .214 | -.195 | -.309* | .443** | -.252 | -.318* | .098 | .360* | -.150 |
| Extraversion | .338** | .122 | .179 | -.203 | -.141 | .334* | -.225 | -.292* | .021 | .336* | -.042 |
| Agreeableness | .210 | -.177 | .216 | -.258 | .122 | .203 | .146 | .153 | .023 | -.023 | -.010 |
| Conscientiousness | .110 | .256 | -.122 | .106 | -.214 | .118 | -.061 | -.142 | .222 | .019 | .014 |
| Emotional stability | .272 | .068 | -.104 | -.141 | -.110 | .278 | .068 | -.038 | -.065 | .039 | .014 |
| Openness | .096 | .253 | .039 | .048 | -.253 | .088 | -.169 | -.112 | -.116 | .046 | .095 |
| (lack of) Premeditation | -.086 | -.127 | .202 | -.020 | .086 | -.084 | .080 | .069 | -.060 | .022 | -.035 |
| Urgency | -.106 | -.045 | .109 | -.020 | .045 | -.106 | -.151 | -.070 | .074 | .050 | -.040 |
| Sensation seeking | .242 | .075 | -.015 | -.116 | -.167 | .243 | -.262 | -.187 | .021 | .251 | -.176 |
| (lack of) Perseverance | -.109 | -.169 | .184 | -.039 | .124 | -.106 | .117 | .151 | -.122 | -.082 | .092 |
| NISROE | -.169 | .062 | .117 | .216 | -.051 | -.174 | .167 | .170 | -.239 | -.215 | .055 |
| Creativity |  |  |  |  |  |  |  |  |  |  |  |
| CCI | .050 | .222 | -.089 | .096 | -.251 | .060 | -.058 | -.042 | .185 | -.107 | .082 |
| Use of Objects task | .003 | .206 | .014 | .148 | -.190 | .013 | -.124 | -.175 | .036 | .010 | .126 |
| Caption generation task | .048 | .182 | -.116 | .062 | -.224 | .054 | -.005 | .062 | .234 | -.173 | .011 |
